# A new short-term toxicity assay using *Aspergillus awamori *with recombinant aequorin gene

**DOI:** 10.1186/1471-2180-5-40

**Published:** 2005-07-02

**Authors:** Olga Kozlova, Mark Zwinderman, Nick Christofi

**Affiliations:** 1 Institute of Cell & Molecular Biology, The University of Edinburgh, King's Buildings, Edinburgh, EH9 3JL, UK; 2 Pollution Research Unit, Napier University, Merchiston Campus, Edinburgh, EH10 5DT, UK; Presently, Surfactant Technologies Ltd., C/o Avecia Fine Chemicals,, Grangemouth, FK3 8XG, UK; 3 Pollution Research Unit, Napier University, Merchiston Campus, Edinburgh, EH10 5DT, UK; 4 LUTESS Ltd., Orchard Brae House,, Edinburgh EH4 2HG, UK

## Abstract

**Background:**

Most currently available short-term toxicity assays are based on bacterial cells. Therefore there is a need for novel eukaryotic microbial bioassays that will be relevant to higher eukaryotes such as animals and plants. Ca^2+ ^is a universal intracellular signalling molecule found in all organisms from prokaryotes to highly specialized animal cells. In fungi calcium has been demonstrated to be involved in control of many important processes. The recombinant aequorin gene from the jellyfish *Aequorea victoria *responsible for the expression of the Ca^2+^-sensitive aequorin photoprotein has been cloned in the filamentous fungus *Aspergillus awamori*. This has allowed real life monitoring of [Ca^2+^]_c _changes in living fungal cells. When subjected to different physico-chemical stimuli fungal cells respond by transiently changing the concentration of free Ca^2+ ^in the cytosol ([Ca^2+^]_c_) and the pattern of these changes (Ca^2+ ^signature) is specific to each particular stimulus. Therefore it was interesting to investigate whether different environmental toxicants would be able to affect the pattern of [Ca^2+^]_c _changes in a reproducible and dose dependant manner.

**Results:**

Toxicity bioassay has been developed to monitor changes [Ca^2+^]_c _of the recombinant fungus in the presence of toxicants representing heavy metals – Cr^6+ ^and Zn^2+ ^and a phenolic polar narcotic -3,5-DCP. The fungus responds to toxicants by a decrease in the amplitude of [Ca^2+^]_c _response to 5 mM external CaCl_2 _and an increase in Ca^2+ ^final resting levels and recovery time.

**Conclusion:**

A novel toxicity bioassay utilizing eukaryotic cells has been developed based on filamentous fungi transformed with the recombinant aequorin gene. A range of parameters characterising changes in [Ca^2+^]_c _has been identified, e.g. Amplitude, Length of Transient, Final Resting Level and Recovery Time. These parameters can be used to determine the toxicity of a range of chemicals to eukaryotic cells in a 96-well microtitre plate method.

## Background

The widespread use and release of natural and synthetic chemicals into the environment, singly or as complex domestic and industrial effluents, has necessitated the development of rapid and cost effective toxicity tests to protect humans and other biota [1,2]. Short-term and long-term bioassays exist utilising microorganisms, invertebrates and higher plants and animals. The short-term microbial toxicity tests involve bacteria, algae, protozoa and fungi (yeasts). One of the most widely used is the Microtox^® ^proprietary test utilising a natural light emitting marine bacterium, *Vibrio fischeri*. *V. fischeri *is a gram negative free-living luminescent bacterium [3,4] also found in some species of fish and squid within the light organs [5].

Bioluminescence involves the emission of visible light mediated by the luciferin-luciferase enzyme system [6]. Microbial bioluminescence is a branch of the electron transport chain [7] and as electron transport is involved in cell metabolism, any disruption to this system e.g. by the presence of toxins, will have an effect on light output. Luminescence is controlled by the *lux *operon [8] within *V. fischeri*, which produces light at 490 nm [7,9].

Calcium is a well-known second messenger involved in the transduction of different external stimuli and hormonal signals in eukaryotic cells. A great number of studies describing changes in [Ca^2+^]_c _have been done describing the role of calcium in animal and plant cells subjected to different treatments. However, very little work in this area has been done on filamentous fungi due to the lack of both routine and reliable methods for monitoring intracellular free Ca^2+ ^in living fungal cells.

Recently recombinant aequorin gene has been expressed in filamentous fungi [10]. Aequorin is a Ca^2+^-sensitive photoprotein of the jellyfish *Aequorea victoria *(Mr = 21,400) [11]. The protein consists of a single polypeptide chain, apoaequorin, a hydrophobic luminophore, coelenterazine and bound oxygen [12]. Once Ca^2+ ^ions are bound to the three Ca^2+^-binding sites in aequorin, the protein is converted into an oxygenase. The oxygenase catalyses the oxidation of the substrate coelenterazine by the bound oxygen and this results in blue light emission. The amount of luminescence emitted by aequorin is dependent upon free Ca^2+ ^concentration and thus aequorin can be used to report cytosolic Ca^2+ ^([Ca^2+^]_c_). Using aequorin transformed fungi it has been shown that different physico-chemical stimuli result in a transient [Ca^2+^]_c _increases with a unique Ca^2+ ^signature [10].

The general purpose of toxicity testing is to establish the potential impact of chemicals on biota in the environment. The information gained can then be used to manage the treatment or release of chemicals. Whether a substance is toxic or not depends on physico-chemical factors such as pH, temperature and salinity, but most importantly on the test organism used, the concentration of the chemical and the conditions imposed for the test (see e.g. [13]. No single toxicity test can determine the effect of toxicants on all biota because of differences in response by organisms at different trophic levels. Eukaryotic organisms can behave differently to prokaryotes although with the *V. fischeri *test a high correlation has been shown with other bioassays using more complex organisms [14].

In this study we have tested the effect of toxic substances on the dynamics of [Ca^2+^]_c _in the fungus *Aspergillus awamori*, containing aequorin gene that constitutively expresses the Ca^2+^-binding photoprotein aequorin. Data are compared with those obtained using the *V. fischeri *bioluminescence assay. This represents the first study in using recombinant aequorin genes in a filamentous fungus to assess toxicity of aqueous samples.

## Results

All three toxicants tested showed a response (Table 1). Parameters assessed were rise time (characterises the time from application of stimulus to maximum amplitude of the response), amplitude (A), Length of Transient (LT_50 _at the point where A = 1/_2_A_max_), final resting level (level of [Ca^2+^]_c _at the end of the experiment) and recovery time (luminescence integrated from the point when amplitude is maximal to the point when [Ca^2+^]_c _reaches its final resting level at the end of the experiment.

**Figure 1:**
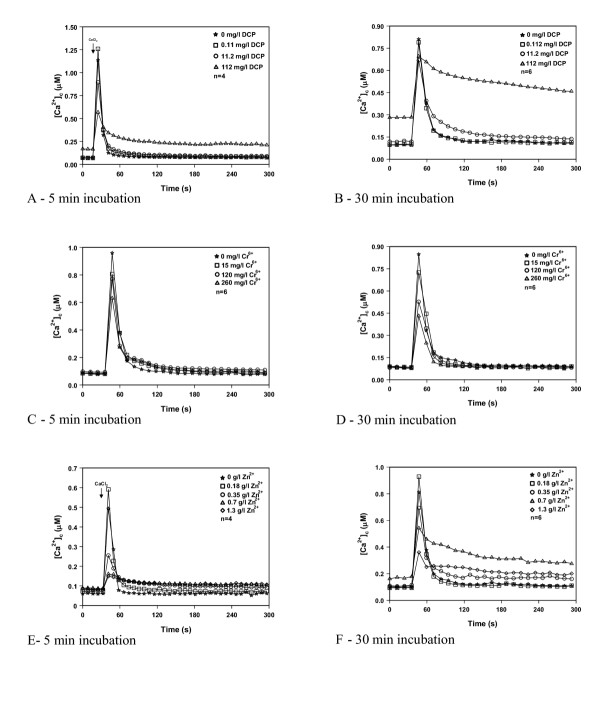
Effect of 3,5-DCP, Cr^6+ ^and Zn^2+ ^on [Ca^2+^]_c _response to the addition of external CaCl_2 _(5 mM). A, C and E show 5 min incubation with the toxicant, B, D F show 30 min incubation with the toxicant.

At low concentration, 3,5-DCP (5 min preincubation) caused a small increase in the amplitude of the [Ca^2+^]_c _response. At high concentrations (11.2 and 112 mgl^-1^) the phenolic inhibited the amplitude of the [Ca^2+^]_c _response at both 5 and 30 min preincubation (Figure 1a &1b; Table 1). The highest concentration of 3,5-DCP (112 mg l^-1^) also increased LT_50 _and rise time (Table 1). At all three concentrations of 3,5-DCP there were elevated final resting levels of Ca^2+ ^and increased recovery times. The percentage increase in the final resting levels and recovery time [15] were used as a quantifying parameter for the analysis of the effect of the phenolic toxicant on [Ca^2+^]_c _(Table 1).

Chromium did not affect (P < 0.5) the final resting levels and recovery time of Ca^2+ ^but had a strong dose dependant inhibition of the amplitude of the [Ca^2+^]_c _response (Figure 1c &1d). Zinc had a combination of these effects on [Ca^2+^]_c _(Figure 1e &1f). It caused a decrease in amplitude of the [Ca^2+^]_c _response as well as an increase in the final resting levels and recovery times at high concentrations (≥ 350 mg l^-1^) (Table 1). Both inhibition of the amplitude and the increase in the final resting levels were dose dependent with the exception of the 1300 mg l^-1 ^Zn^2+ ^at 30 min preincubation. The increase of the above mentioned parameters observed at this concentration was lower than when fungi were treated with 700 mg l^-1 ^of Zn^2+^. This was probably due to aequorin quenching which is known to be caused by the very high concentrations of some heavy metals. At low concentration (180 mg l^-1^) Zn^2+ ^caused an increase in amplitude of the [Ca^2+^]_c _response as well as an increase in the final Ca^2+ ^resting level and recovery time.

Recovery of treated *A. awamori *to basal Ca^2+ ^levels and light dissipation was fairly rapid in systems challenged with Cr^6+ ^for 5 and 30 min. This was not the case for *A. awamori *treated with Zn^2+ ^and 3,5-DCP. In these cases there was protracted recovery in Ca^2+ ^content at higher toxicant challenges with both 5 and 30 min incubation.

**Figure 2:**
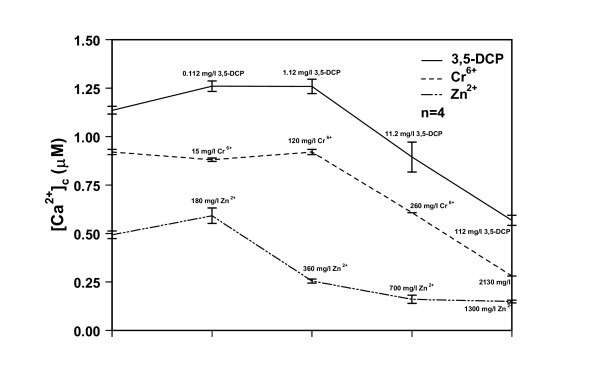
Effect of 3,5-DCP, Cr^6+ ^and Zn^2+ ^on amplitude of [Ca^2+^]_c _in *A. awamori *5 minute incubation.

**Figure 3:**
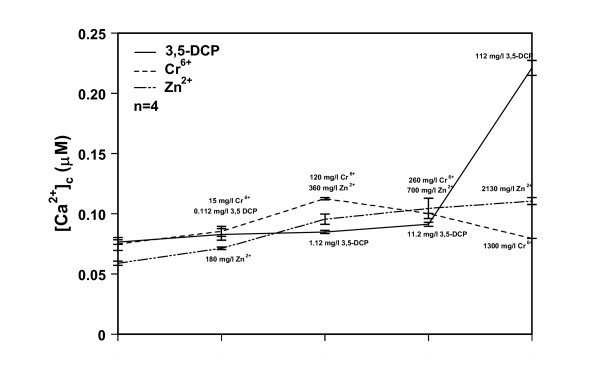
Effect of 3,5-DCP, Cr^6+ ^and Zn^2+ ^on final resting level of [Ca^2+^]_c _in *A. awamori *5 minute incubation.

IC_50 _is one standard parameter calculated in toxicity studies and was chosen in this study to determine differences in values obtained by the fungal and bacterial luminescence bioassays. In order to calculate IC_50 _values for all toxicants it was decided to utilise the amplitude of [Ca^2+^]_c _changes in the fungal cell after challenge with different concentrations of toxicant. Examples of dose response curves are presented in Figures 2, 3. Calculated IC_50 _values for the 5 and 30 min toxicant pre-incubations with *A. awamori *are presented in Table 2. The 30 min IC_50 _for DCP, Cr^6+ ^and Zn^2+ ^are 36.7, 167.8 and 549.7 mg l^-1 ^respectively. These can be compared to values for the *V. fischeri *test of results of 3.6, 13.95 and 0.44 mg l^-1 ^for the same toxicants tested over a 30 min incubation period.

## Discussion

In this paper data have been presented on the response of *A. awamori *to different toxicant concentrations by examining changes in Relative Light Units (RLU) and [Ca^2+^]_c _in the presence of an external CaCl_2 _concentration of 5 mM. This study differs from that of Torrecilla *et al. *[16] which examined changes in intracellular Ca^2+ ^of the cyanobacterium *Anabaena *able to express apoaequorin constitutively when subjected to heat and cold shock. It is also the first study, as far as we know, examining responses of aequorin to toxicants in a filamentous fungus. The concentrations of Zn^2+^, Cr^6+ ^and 3,5-DCP used in the experiments to assess toxicity were based on experience with *V. fischeri *bioassays, through range finding and interest in observing effects at low and high concentrations. Initially, the effect of each of these three chemicals on [Ca^2+^]_c _was examined. The [Ca^2+^]_c _response was not significantly different from that obtained with the control solution (data not shown). A second approach studied the effect of preincubation of the fungus with toxicants on [Ca^2+^]_c _response to external CaCl_2_.

There is no doubt that aequorin transformed *A. awamori*, using the protocol developed, responds to toxic chemical challenge in a reproducible way. The aequorin system has more parameters which can be assessed than other bioassays i.e. final [Ca^2+^]_c _resting level, recovery time and amplitude of [Ca^2+^]_c_/RLU following chemical challenge for 5 or 30 min.

*A. awamori *has been shown to respond to organic and inorganic compounds by a decrease in the amplitude of [Ca^2+^]_c _response to external CaCl_2 _with increasing toxicant concentration. Zn^2+ ^and DCP also affected the final Ca^2+ ^resting levels and recovery times. In the case of 30 min preincubation with either 112 mg l^-1 ^DCP or 700 mg l^-1 ^Zn^2+^, the final [Ca^2+^]_c _resting levels remained significantly higher than after 5 min preincubation with these toxicants indicating a greater toxicity to the fungus on longer contact with the toxicant. This is not an unusual response even in the *V. fischeri *bioassay where it is generally observed that toxicity increases (IC_50 _values decrease) with longer incubation times. IC_50 _values calculated using amplitude changes of [Ca^2+^]_c _showed decreased toxicity to Zn^2+ ^at longer preincubation times. This needs further analytical consideration to determine toxicity values of relevance.

The proprietary Microtox bioassay and other bacterial bioluminescence methods, including that used in the present study, which do not involve genetically modified bacteria, utilise *Vibrio *(e.g. *V. fischeri*) and related *Photobacterium *species. Acute toxicity tests utilising such luminescent bacteria can underestimate the toxicity of chemicals and Backhaus *et al. *[17] showed that more reliable toxicity estimates can be obtained through the use of long-term toxicity testing with the same organisms. It may be prudent to test the response of *A. awamori *using longer preincubation times with toxicants e.g. > 24 h, prior to monitoring Ca^2+ ^homeostasis. This may increase the sensitivity of the bioassay. It must be remembered however that for one of the toxicants (Zn^2+^), a decrease in sensitivity was evident at 30 min compared to the result at 5 min. There was also evidence of toxicity recovery through adaptation with increased incubation. This may be due to acquired resistance by the organism through synthesis of metal-binding proteins (metallothioneins) or constituents in the growth medium removing/immobilising the metal (e.g. EDTA, phosphate precipitation). This needs to be examined in the fungal bioassay.

It is interesting to compare toxicity data obtained with *A. awamori *with those obtained with the *V. fischeri *bioassay. The toxicity values for the fungus are higher than those of the bacterial test indicating lower bioassay sensitivity with the parameter used to calculate the IC_50 _values. For example, the IC_50 _values for the 30 min Zn^2+ ^incubations were approximately 3 orders of magnitude lower for *V. fischeri*. Such insensitivity to Zn^2+ ^has been observed with an ATP luminescence assay [18]. The IC_50 _results with Cr^6+ ^(30 min preincubation) were 400 mg l^-1^, 12 times higher than in the bacterial biosensor. This value is the same order of magnitude as the 15 min Microtox assay (339.6 mg l^-1^) carried out by Codina *et al. *[19]. Indeed, the use of *V. fischeri *in various proprietary tests (LumisTox, Microtox, ToxAlert etc) have been shown to exhibit differences in sensitivity to different toxicants [20]. It is, therefore, difficult to categorically state that *V. fischeri *is more sensitive to Cr^6+ ^than *A. awamori *particularly when different incubation periods are used in bioassays affecting any ultimate IC_50 _value. It should be also noted that since the aequorin test is based on changes in [Ca^2+^]_c_, toxic chemicals often exert an increase in [Ca^2+^]_c _thus affecting IC_50 _calculations. The calculation of IC_50 _for this fungal bioassay may not be appropriate. Other parameters such as LT_50_, rise time, final Ca^2+ ^resting levels and recovery time would provide ideal candidate parameters, but more tests and comparisons with other bioassays are needed. The aequorin bioassay can also generate up to 15 parameters to assess the effect of toxicants due to the complex pattern of [Ca^2+^]_c _changes [21]. These parameters can be further used to create a profile for toxicants with specific modes of action [15]. With different parameters available for analysis in the aequorin test it could be useful to assess the toxicity by calculating the NOEC (no observable effect concentration) or a nominal IC_10 _value [22].

Codina *et al. *[19] also used a yeast bioassay to test metal toxicity. The eukaryotic yeast was found to be less sensitive to metals than prokaryotic organisms including *Vibrio *and *Pseudomonas *species. An IC_50 _value of 549.1 mg l^-1 ^was obtained for Zn^2+ ^using the yeast assay [19] which is comparable to some of the values obtained with *A. awamori *in our study. The yeast was slightly more sensitive to Cr^6+ ^(30.9 mg l^-1^) than the filamentous *A awamori *but this is not unusual among members of the same trophic level (see [19]. Recent chronic toxicity studies have been using wild-type and genetically modified mutants of the yeast *Saccharomyces cerevisiae *[23]. EC_50 _values of >1000 mg l^-1 ^were determined for Zn^2+ ^and 1.7-4.79 mg l^-1 ^for Cr^6+ ^indicating variability in assays that rely on conditions imposed including exposure time, species tested and criteria used in the final assessment.

An interesting observation is the enhanced stimulation of light output in some systems with a low concentration of toxicant. This is seen in the 5 min treated systems using 0.11 mg l^-1 ^DCP (Figure 1a), 180 mg l^-1 ^Zn^2+ ^(Figure 1e) and also in the 30 min treated system with 180 mg l^-1 ^Zn^2+ ^(Figure 1f). This stimulation, referred to as hormesis, is a common occurrence in toxicity bioassays [13] and is often observed in our laboratory using the *V. fischeri *bioassay.

It is not clear based on these preliminary observations what causes Ca^2+ ^and light retention. In the case of DCP it may be that this polar narcotic is affecting membrane permeability and the transport of Ca^2+ ^out of the cytosol (Ca^2+ ^ATPase and efflux of Ca^2+ ^via e.g. Ca^2+^/H^+ ^antiporters) and hence, a delay in RLU dissipation. The metals may be competing with the transport mechanisms in membranes. The metals thus act by interacting with physiological ions affecting transport and its concomitant effect on light output.

Most of the organic chemicals discharged to the environment exert a narcotic effect on biota. This is either Type I (non-polar narcosis) or Type II (polar narcosis). It would be interesting to utilise *A. awamori *to test whether non-polar and polar narcoses operate in the fungus and whether these can be predicted by QSAR (Quantitative Structure Activity Relationships). Cronin and Schulz [24] showed, using QSAR, that non-polar narcosis occurs in *V. fischeri *but they could not validate polar narcosis in the organism.

There is no doubt that a comprehensive testing of metals and organic compounds needs to be carried out to assess the value of *A. awamori *as a toxicity sensing tool both in systems using pure chemicals and those involving real samples (e.g. complex effluents and soil water matrices). Toxicant preincubation time and effect on fungus response would be an important factor to test fully.

## Conclusion

The current paper presents the proof-of-concept study of the novel toxicity bioassay based on using filamentous fungi transformed with the recombinant aequorin gene. The research conducted has shown that the recombinant aequorin method can be used as a novel eukaryotic toxicant biosensor. It offers more parameters which can be readily analysed than the traditionally used bacterial biosensors. The fungal aequorin biosensor responded to toxic substances (3,5-DCP, Zn^2+ ^and Cr^6+^) by a decrease in the amplitude of the [Ca^2+^]_c _response to 5 mM CaCl_2 _and an increase in the [Ca^2+^]_c _resting levels (Zn^2+ ^and 3,5-DCP). Preliminary IC_50 _values obtained with the fungal aequorin system, and based on the inhibition of the [Ca^2+^]_c _amplitude in response to external CaCl_2 _treatment, indicate that it is less sensitive for detecting 3,5-DCP, Zn^2+ ^and Cr^6+ ^than the *V. fischeri *luciferase system. However, it is obvious that the changes in amplitude is not the most suitable parameter for calculating IC_50 _and more research are needed to determine the best possible set of parameters to be used together with the aequorin bioassay.

## Methods

### Chemicals

Unless otherwise stated all the chemicals used were from Calbiochem-Novabiochem (UK) Ltd. (Nottingham, UK) or Sigma (Dorset, UK).

### Organisms and media

Experiments were carried out with the strain of *A. awamori *66A, which has been transformed with the recombinant aequorin gene [10]. All media and salt solutions were made up in distilled H_2_O and sterilised by autoclaving at 121°C at 15 psi for 20 min prior to use. *A. awamori *cultures were grown in liquid Vogel's medium with 1% sucrose (VS medium [25]).

A method of growing A. awamori cultures for in vivo luminometry was employed utilising static liquid culture in 96-well plates. Twelve ml of sterile VS medium was inoculated with 1 × 10^5 ^spores per ml. Coelenterazine (Biosynth AG, Staad, Switzerland) was dissolved in methanol (MeOH) to a final concentration of 2.5 μM. The final MeOH concentration was not more than 0.1%, which has been shown not to affect spore germination or hyphal growth. Using a 12-channel pipette (Anachem, Luton, UK), 100 μl of the inoculated medium was added to each well, and the plate covered with a microplate lid (Labsystems, Helsinki, Finland). Cultures were incubated in a humidity chamber in the presence of free water at 30°C for 24 h.

Luminometry was performed using an EG & G Berthold (Bad Wildbad, Germany) LB96P Microlumat luminometer. The luminometer was designed to measure luminescence using the flat-bottomed 96-well microtitre plates from EG&G Berthold or Dynex Technologies Inc. (Chantily, UK). Two 100 μl built-in injectors allowed stimulation of samples.

The luminometer measures light emission in relative lights units (RLU). To convert RLU into [Ca^2+^]_c _concentrations the following empirically derived equation was used:

pCa = 0.332588 (-log k)+5.5593,

where k = luminescence counts s^-1^/ total luminescence counts [26].

Total luminescence is measured as an integral of all luminescence up to complete aequorin discharge. Aequorin was discharged by adding 2M CaCl_2 _in 20% ethanol to the wells. Ethanol was used as a permeabilising agent. Conversion of RLU into [Ca^2+^]_c _concentration was made using a Macro developed in Excel 7.0 by Mark Knight (University of Oxford).

Three toxicants were tested: 3,5-DCP, ZnSO_4 _and K_2_Cr_2_O_7_. All toxicants were added 5 min and 30 min before treatment with 5 mM external CaCl_2 _in a total volume of 25 μl VS medium. 4-6 replicates were performed for each treatment. All the experiments were performed in triplicate. IC_50 _values were calculated only for the amplitude of the response.

*Vibrio fischeri *bioassays utilised cultures of the organism grown in *Photobacterium *Broth (PB) at 37°C until optimum light output. A 100 μl volume of *V. fischeri *suspension was added to each of the 96 wells in a microtitre plate. During the experiment a 100 μl volume of NaCl solution (giving a final concentration of 2% NaCl in the test) containing the test substance in the appropriate concentration was added to the wells. For each measurement a control consisting of 6 wells with *V. fischeri *to which the NaCl solution was added. Chemicals were tested in five different concentrations and each concentration was tested in three replicates. Plates were measured in an Anthos Lucy 1 luminometer. Measurements were made over a period of 30 min. IC_50 _values were calculated for 5 and 30 min incubations. Recording of data started immediately after addition of the test solutions to the wells containing the culture. The standard proprietary Microtox bioassay was also carried out [27].

## Authors' contributions

OK conceived the study, designed and developed aequorin bioassay, carried out the tests and has been involved in the writing of the article.

MZ has designed the microbial part of the testing and participated in carrying out the fungal tests.

NC helped design the study and participated in writing of the article.

All authors read and approved the final manuscript.

## Figures and Tables

**Table 1:** Detailed analysis of the effects of preincubating cultures of *A. awamori *with 3,5-DCP, Cr^6+ ^and Zn^2+ ^on [Ca^2+^]_c _response to 5 mM external CaCl_2_

Toxicant		Features of [Ca^2+^]_C _Signatures					
		A	LT_50_	FRL (%) 5 min pre-incubation	RT (%) 5 min pre-incubation	FRL (%) 30 min pre-incubation	RT (%) 30 min pre-incubation

3,5-DCP	0.112	↑	-	111 ± 8.4 (↑)	111 ± 8.4 (↑)	106 ± 7.6 (-)	98 ± 1.3 (-)
(mgl^-1^)	11.2	↓	-	120 ± 4.8 (↑)	125 ± 4.6 (↑)	127 ± 29.7 (-)	134 ± 31.9 (↑)
	112	↓	↑	289 ± 17.6 (↑)	292 ± 6.6 (↑)	416 ± 23.2 (↑)	416 ± 16.6 (↑)
Cr^6+^	15	↓	-	-	-	-	-
(mgl^-1^)	120	↓	-	-	-	-	-
	260	↓	-	-	-	-	-
Zn^2+^	180	↑	-	106 ± 9.2(-)	127 ± 4.6 (↑)	97 ± 6.9(-)	92 ± 1.9(↓)
(mgl^-1^)	350	↓	-	153 ± 13.6(↑)	167 ± 14.4(↑)	149 ± 11.4(↑)	143 ± 10.2(↑)
	700	↓	↑	164 ± 30.7(↑)	179 ± 26.9(↑)	245 ± 16.5(↑)	261 ± 9.6(↑)
	1300	↓	↑	169 ± 14.8(↑)	182 ± 10.4(↑)	169± 12.6(↑)	176 ± 16.5(↑)

Note: – no effect compared with untreated control), ↑ increase in parameter, ↓ decrease in parameter, A – amplitude, LT_50 _– Length of transient, FRL – Final Ca^2+ ^resting level, RT – recovery time. A and LT_50 _are shown only for 5 min incubation. Results represent mean ± SD. The % values represent the absolute value of the parameter not the relative increase compared with control. N = 6

**Table 2:** IC_50 _values (mgl^-1^) for 3,5-DCP, Cr^6+ ^and Zn^2+ ^using *V. fischeri *and *A. awamori*, (based on amplitude of [Ca^2+^]_c_)

Treatment	*V.fischeri*		*A. awamori*	
Test duration or preincubation time	IC_50 _(5 min)	IC_50 _(30 min)	IC_50 _(6 min)	IC_50 _(30 min)

3,5-DCP	3.62	3.13	46.7	36.7
Cr^6+^	29.9	13.95	400.1	167.9
Zn^2+^	95.56	0.44	237.2	549.7

Note: N = 6
